# TFF3 as a Diagnostic Biomarker in Kidney Transplant Patients

**DOI:** 10.3390/ijms241511925

**Published:** 2023-07-25

**Authors:** Karolina Rogulska, Iwona Wojciechowska-Koszko, Barbara Krasnodębska-Szponder, Paweł Kwiatkowski, Paulina Roszkowska, Barbara Dołęgowska, Karolina Łuczkowska, Bogusław Machaliński, Danuta Kosik-Bogacka

**Affiliations:** 1 Department of Diagnostic Immunology, Pomeranian Medical University in Szczecin, Powstańców Wielkopolskich 72, 70-111 Szczecin, Poland; karolina.rogulska@pum.edu.pl (K.R.);; 2 Immunology Laboratory, Independent Public Clinical Hospital No. 2, Powstańców Wielkopolskich 72, 70-111 Szczecin, Poland; 3 Department of Laboratory Medicine, Pomeranian Medical University in Szczecin, Powstańców Wielkopolskich 72, 70-111 Szczecin, Poland; 4 Department of General Pathology, Pomeranian Medical University in Szczecin, Powstańców Wielkopolskich 72, 70-111 Szczecin, Poland; 5 Independent Laboratory of Pharmaceutical Botany, Pomeranian Medical University in Szczecin, Powstańców Wielkopolskich 72, 70-111 Szczecin, Poland

**Keywords:** transplantation, biomarkers, intestinal trefoil factor 3

## Abstract

Intestinal trefoil factor 3 (TFF3) is a protein secreted by many cell types, and its serum and urine levels vary in patients with kidney disease. Therefore, the present study aimed to determine the diagnostic value of TFF3 in allogeneic kidney transplant patients included in the one-year follow-up. To analyze the influence of the diagnostic method used, we studied the type of biological material and the time elapsed since renal transplantation on the parameter’s value. The study also aimed to investigate the relationship between TFF3 levels and creatinine and estimated glomerular filtration rate (eGFR) values in the serum and urine of the patients studied. The study used blood and urine samples from adult patients (*n* = 19) 24–48 h, 6 months, and 12 months after kidney transplantation. We collected one-time blood and urine from healthy subjects (*n* = 5) without renal disease. We applied immunoenzymatic ELISA and xMap Luminex flow fluorimetry to determine TFF3 in serum and urine. There was a significant difference in TFF3 levels in the serum of patients collected on the first one or two days after kidney transplantation compared to the control group (determined by ELISA and Luminex) and six months and one year after kidney transplantation (ELISA). We observed a correlation between creatinine concentration and urinary TFF3 concentration (ELISA and Luminex) and a negative association between eGFR and urinary (ELISA) and serum (Luminex) TFF3 concentration in patients on the first and second days after kidney transplantation. We noted significant correlations between eGFR and TFF3 levels in the serum and urine of patients determined by the two methods six months and one year after transplantation. In women, we observed that urinary TFF3 concentration increased significantly with increasing creatinine and that with increasing eGFR, urinary TFF3 concentration determined by two methods decreased significantly. In the present study, the choice of diagnostic method for the determination of TFF3 in serum and urine significantly affected the concentration of this biomarker. The values of this parameter determined by ELISA were higher than those assessed using the Luminex assay. Based on the presented results, we can conclude that TFF3 has great potential to monitor renal transplant patients. Determination of this protein in parallel with creatinine and eGFR levels in serum and urine may provide helpful diagnostic information.

## 1. Introduction

Indirect assessment of renal damage in patients after allogeneic kidney transplantation is mainly based on evaluating values of classical laboratory parameters, including serum electrolytes, urea, and creatinine, with an estimation of estimated glomerular filtration rate (eGFR) and a general urinalysis, which is insufficient. Therefore, the panel of these tests should be expanded to include new parameters, including protein biomarkers [1]. A biomarker of renal injury should be an indicator that can be measured and assessed as a component of a pathogenic process, biological process or pharmacological response [2].

Intestinal trefoil factor 3 (TFF3) is a member of the human trefoil factor family, along with the peptides TFF1 and TFF2 [3]. This protein is mainly secreted by mucosal cells of the small and large intestines [4]. It allows it to act as a biomarker in ulcerative colitis and correlates well with acute phase protein levels [5]. In addition, this protein may participate in glucose metabolism [6]. TFF3 is also secreted by nerve cells and regulates learning processes [7]. This peptide has neuroprotective effects, as it extinguishes caspase-3 activity, which damages microglial cells [8]. TFF3 also has anti-apoptotic and pro-proliferative functions and is thought to contribute to the progression of solid tumors [9]. In addition, TFF3 may influence the metastasis of cancer cells in epithelial tissues [10]. The effect of TFF3 on the regenerative capacity of the mucosa has led to ongoing attempts to use this protein in therapy [11]. We suggest that TFF3 expresses in all mucus-secreting tissues, including renal tubules.

The intestinal trefoil factor can be used as a biomarker in patients with kidney damage [12]. The results show that serum levels of TFF3 are significantly higher in patients with chronic kidney disease (CKD) than in controls. In addition, we observed that the level of this protein is higher in patients with CKD than in those with other lifestyle diseases [13]. TFF3 levels also increased in the urine of patients with worsening chronic kidney disease, and in combination with the presence of microalbuminuria, this protein may be a predictor of a worse prognosis [14]. The Food and Drug Administration (FDA) and the European Medicines Agency (EMA) have recognized the determination of urinary TFF3 levels as a specific and sensitive biomarker for monitoring drug-induced kidney injury [15]. TFF3 has also been analyzed as a marker of the autoimmune process. Yan et al. [16] noted that plasma TFF3 levels were higher in systemic lupus erythematosus (SLE) patients with nephritis than in those with SLE without renal lesions. In addition, levels of this protein correlate with clinical features of dysfunction in lupus nephritis. TFF3 levels may increase in children with congenital renal and urinary tract abnormalities and may predict worsening renal function [17]. It had high serum levels of this protein immediately after kidney transplantation and a subsequent decrease, irrespective of delayed graft function (DGF) [18]. The role of TFF3 as a marker of renal allograft rejection is not yet well understood. Therefore, the present study aimed to determine the diagnostic value of TFF3 in allogeneic kidney transplant patients included in the one-year follow-up period and to analyze the influence of the diagnostic method used, the type of biological material and the time elapsed since kidney transplantation on the value of the parameter studied. In addition, we analyzed the relationship between TFF3 levels and creatinine and eGFR values in the patients studied.

## 2. Results

Table 1 shows TFF3 levels determined in the serum and urine of control subjects and patients one to two days, six months, and one year after kidney transplantation. The concentration of this protein was highest in the serum and urine of patients one day after kidney transplantation and then decreased six months and one year after surgery. Only for the Luminex urinary TFF3 assay were concentrations highest in patients one year after kidney transplantation. We found much lower TFF3 concentrations in the serum and urine of control subjects. In renal transplant patients, creatinine concentrations decreased with time while eGFR values increased.

We used the nonparametric Mann-Whitney U test (Table 2) to compare patients’ serum and urine TFF3 concentration values at different times after kidney transplantation obtained using ELISA and Luminex with the control group. There was a significant difference in the concentration of TFF3 determined in patients’ serum collected one to two days after kidney transplantation compared to the control group determined by ELISA (*p* = 0.001) and Luminex (*p* = 0.004). Additionally, TFF3 levels in patient serum were determined using ELISA six months (*p* = 0.009) and one year (*p* = 0.013) after kidney transplantation.

There was a correlation between creatinine levels and TFF3 levels in urine collected from patients on the first and second days after renal transplantation, as determined by ELISA (*p* = 0.022) and Luminex (*p* = 0.006) (Table 3). In women after renal transplantation, we observed that urinary TFF3 levels determined by ELISA (*p* = 0.013) and Luminex (*p* = 0.030) increased significantly with increasing creatinine (Table 4). Significant correlations between creatinine levels and TFF3 levels in serum and urine determined by the two methods were noted in all patients studied six months and one year after renal transplantation.

The correlation analysis between eGFR and TFF3 concentration was in line with the relationships discussed above (Table 3). On the first and second days after renal transplantation, all study patients had a negative correlation between eGFR and urine TFF3 concentration determined by ELISA (*p* = 0.004) and serum TFF3 concentration determined by Luminex (*p* = 0.004). In the female renal transplant patients studied, the urinary TFF3 concentration determined by the two methods decreased significantly with increasing eGFR. In all study patients six months and one year after transplantation, significant correlations were observed between eGFR and TFF3 concentrations in both serum and urine, determined by two methods.

There was a significantly higher concentration of TFF3 (*p* < 0.0001) in the serum of renal transplant patients tested at three time points combined using ELISA (Me = 20.35 ng/mL) relative to the TFF3 value determined by Luminex (Me = 6.02 ng/mL). It was confirmed in a detailed analysis one to two days, six months, and one year after kidney transplantation. Serum TFF3 levels in renal transplant patients assessed using ELISA were significantly higher one to two days (*p* < 0.0001), six months (*p* < 0.0001), and one year after renal transplantation (*p* < 0.0001) relative to values obtained using the Luminex method. When TFF3 levels were averaged in the urine of renal transplant patients collected at the three time points together, we found significantly higher levels of this biomarker (*p* < 0.0001) determined by ELISA (Me = 122.33 ng/mL) relative to values determined by Luminex (Me = 35.39 ng/mL). Detailed analyses separately for each of the three intakes also confirmed it.

**Figure 1:**
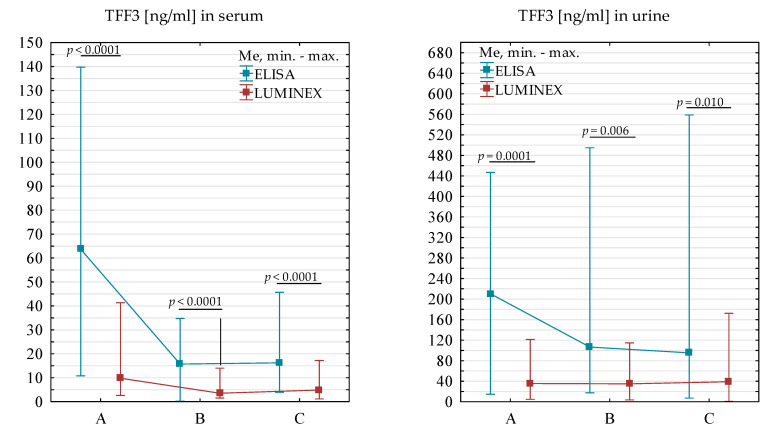
Comparison of TFF3 values in serum and urine of patients collected one to two days (**A**), six months (**B**), and one year (**C**) after kidney transplantation using ELISA and Luminex methods.

The concentration of TFF3 in the urine of patients determined by ELISA was significantly higher one to two days (*p* < 0.0001), six months (*p* = 0.006), and one year (*p* = 0.010) after kidney transplantation compared to the values of this biomarker determined by Luminex (Figure 1). The differences between the values obtained by the two methods may be due to the test itself. ELISA is a technique to detect relative mass values for naturally occurring human TFF3, but Luminex is used to evaluate many parameters at once. Along with this marker, other proteins are also found in the standard cocktail. The same diluent and optimal pH must be used for all tested parameters, which may cause discrepancies in the values obtained between methods.

Due to the large scatter in the data, the change in values between the measurements was additionally analyzed. This analysis showed differences between the methods only in the measurements on the first day after transplantation and after six months and between measurements on the first day after transplantation and one year after transplantation in serum (Table 5).

The relationship between glucose and TFF3 was also checked by Spearman’s rank method six months and a year after kidney transplantation by ELISA and Luminex methods, but no significant correlations were found (*p* > 0.05).

## 3. Discussion

The results presented here extend the knowledge regarding the usefulness of testing serum and urine TFF3 levels in renal transplant patients. It appears that analysis of this parameter may reflect renal function after transplantation.

Various cells synthesis TFF3 and have many functions, including involvement in wound healing, mucosal protection, cell proliferation and migration. However, the role of TFF3 in these processes is not fully understood. Clinical and experimental findings indicate that TFF3 is also involved in many pathological processes, including mucosal disorders and cancer [19,20]. Increased expression of TFF3 has been observed in some cancers, including breast, lung, liver, prostate, gastric, and endometrial cancers [21,22,23,24,25,26,27]. This peptide has potential value as a biomarker, including cancer metastasis [28,29].

TFF3 is synthesized in the urinary tract epithelia, mainly the proximal and distal tubules and collecting ducts [30]. Elevated urinary TFF3 levels have been found in people of African descent, patients with diabetes and those taking blood pressure-lowering drugs [31]. We concluded that TFF3 might influence the regenerative capacity of the kidney, possibly through restitution after injury, effects on differentiation, or both [31]. Tanaka et al. [32] found that increased TFF3 mRNA expression in renal biopsy specimens from patients with tubulointerstitial fibrosis in IgA nephropathy (IgAN) was associated with increased urinary TFF3 levels and that examination of urinary TFF3 levels may reflect interstitial tubular fibrosis in IgAN patients. We found elevated serum and urine TFF3 levels in patients with CKD, which may be due to the secretion of this peptide by damaged renal tubular epithelial cells [13]. Increased levels of TFF3 have been associated with excess mortality risk, which traditional markers of kidney disease may overlook. Endre [18] described a study by Pianta et al. (unpublished data), which noted an increase in TFF3 levels in patients (*n* = 75) after transplantation and a subsequent decrease, irrespective of the presence of DGF. The present study confirmed this relationship and showed that serum TFF3 levels determined by ELISA decrease significantly after renal transplant surgery. In contrast, no such relationship was shown in serum as determined by the Luminex method or in urine by both methods. In contrast, Pianta et al. [33], based on an analysis of serum and urine results from kidney recipients (*n* = 81), concluded that urinary TFF3 concentration testing is not a promising biomarker for the early diagnosis of delayed graft function.

The study presented here extends the knowledge of this biomarker in renal function after transplantation, as seen by the significant correlations between TFF3 and creatinine and eGFR at different times. We found the choice of diagnostic method for the determination of TFF3 in serum and urine to have a significant effect on the concentration of this biomarker. The values of this parameter determined with ELISA are higher than those assessed using the Luminex assay.

There are several limitations worth noting in this analysis. A small sample size and the single center approach suggest the need for validation in larger, multi-center populations. It would be judicious to plan a biopsy prior performed at time points corresponding to blood and urine collections. Furthermore, our findings need to be validated through other studies to ensure consistency of observed associations and their subsequent impact on clinical trial design. Monitoring patients for only 24 h, 6 and 12 months after kidney transplantation may not have captured all variables which contribute to ongoing renal injury in this group. However, a notable strength of the study is the prospective design which enabled access to urine output and serum creatinine values for 24 h, 6 and 12 months after kidney transplantation in all patients. Thus, it is likely that this marker will show clinically relevant performance. Further research efforts are certainly needed for the pursuit of data on each patients clinical course, such that it is understood whether or not the TFF3 levels that are being obtained might possibly also reflect other recent events in the patients clinical course. 

Based on the results of our and other authors’ studies, TFF3 is a promising marker for monitoring the status of renal transplant patients; however, these data should be approached with caution, and further studies are needed.

## 4. Materials and Methods

We conducted the study between 2018 and 2022. The study included adult kidney transplant patients from deceased donors in the region of northwestern Poland. They were patients of the Transplant Clinic of the Independent Public Clinical Hospital No. 2 PUM in Szczecin. The exclusion criterion for the study was the recipient’s age below 18 years, and the inclusion criterion was preserved graft function one year after surgery. The study group comprised 19 patients, including nine women and 10 men, aged 26 to 71 years (mean age 51.9 ± 12.1 years) and weighing 61 to 114 kg (mean 78.32 ± 13.33 kg). Their mean time on dialysis was 2.79 ± 3.60 years (0 to 16 years). Demographic data of the study group are presented in Table 6. We collected fasting blood from the patients in the morning and urine at intervals according to the schedule: 1–2 days, 6 months, and 12 months. Samples were collected at these time points to determine the dynamics of changes in concentrations of selected biomarkers in short- and long-term post-transplant evaluation. We collected 57 blood samples and 57 urine samples in the study group. In addition, analogous material was also collected once from five healthy subjects (three women and two men) aged between 28 and 44 years (mean subject age 32.6 ± 6.69 years) and weighing between 58 and 90 kg (mean 75.6 ± 14.47 kg) without renal disease, who constituted the control group. Table 7 shows the demographic dataof the control group. Characteristics of patients in the study and control groups are presented in Table 8.

The Bioethics Committee of the Pomeranian Medical University in Szczecin (resolution no. KB-0012/114/12) approved the study. The study was conducted following the Declaration of Helsinki.

We used two methods to determine TFF3 concentrations: enzyme-linked immunosorbent assay immunoenzymatic (ELISA) and xMap Luminex flow fluorimetry. Both assays were from R&D Systems (Minneapolis, MN, USA): the Human TFF3 Quantikine ELISA Kit and the Human Kidney Biomarker Premixed Magnetic Luminex^®^ Performance Assay. We performed the assays according to the protocols provided by the manufacturer. Serum creatinine was determined using a colorimetric assay based on the Jaffé method on a Cobas C 501 instrument from Roche Diagnostics (Mannheim, Germany), while eGFR was calculated according to the CKD-EPI formula.

Statistical results were analyzed using Statistica 13.3 (Statistica PL, StatSoft). We examined the distribution of the data using the Shapiro-Wilk test, taking into account the division into test and control groups and separately for the values of the variables obtained on the first day, six months, and one year after renal transplantation. A nonnormal distribution characterized the variables assessed, so data were presented in tables and graphs in the form of the median, minimum, and maximum values and lower and upper quartiles, and the tests used in the analyses were nonparametric.

## 5. Conclusions

In conclusion, TFF3 in serum and urine may be a promising biomarker for diagnosing renal function and prognosis, mainly on the first and second days after kidney transplantation. Simultaneous determination of this biomarker and creatinine and eGFR levels in the patient’s serum and urine may provide helpful diagnostic information. Furthermore, large-scale studies are warranted to investigate the diagnostic and prognostic value of serum and urine TFF3 levels in renal transplant patients.

## Figures and Tables

**Table 1:** Creatinine, estimated glomerular filtration rate (eGFR), and trefoil factor 3 (TFF3) concentrations in serum and urine of study patients collected one to two days, six months, and one year after kidney transplantation were assessed using ELISA and Luminex (Med., median; Q_1_, lower quartile; Q_2_, upper quartile).

Group	Time after Kidney Transplantation	Creatinine (in mg/dL) Med (Q_1_–Q_2_)	eGFR (in mL/min/1.73) Med (Q_1_–Q_2_)	Concentration of TFF3 (in ng/mL)			
				ELISA		Luminex	
				Serum Med (Q_1_–Q_2_)	Urine Med (Q_1_–Q_2_)	Serum Med (Q_1_–Q_2_)	Urine Med (Q_1_–Q_2_)
control		0.78 (0.65–0.93)	94 (92–112)	7.84 (6.11–8.17)	89.26 (79.72–90.78)	3.18 (2.10–3.86)	31.95 (23.88–37.78)
patients after kidney transplantation	One to two days	4.05 (2.58–5.94)	13 (9–21)	63.65 (31.51–82.58)	210.4 (66.62–273.9)	9.79 (8.24–21.46)	35.39 (14.85–72.29)
	Six months	1.44 (1.14–1.53)	52 (43–68)	15.73 (12.76–24.89)	106.5 (43.38–268.1)	3.52 (2.71–6.78)	34.90 (16.13–76.40)
	One year	1.32 (1.18–1.58)	53 (47–63)	16.22 (10.54–20.94)	95.16 (54.80–290.8)	4.87 (2.76–6.97)	39.11 (19.64–83.27)

**Table 2:** Comparison of TFF3 values in serum and urine of patients of the study group at different times after kidney transplantation and the control group obtained using ELISA and Luminex (SG, study group; CG, control group; A, one to two days after kidney transplantation; B, six months after kidney transplantation; C, one year after kidney transplantation; U, Mann-Whitney test value for small-size groups; *p*, significance level *p* = 0.05).

TFF3 Level	Time after Kidney Transplantation											
	One to Two Days				Six Months				One Year			
	Sum of Ranks SG	Sum of Ranks CG	U Mann-Whitney		Sum of Ranks SG	Sum of Ranks CG	U Mann-Whitney		Sum of Ranks SG	Sum of Ranks CG	U Mann-Whitney	
			U	*p*			U	*p*			U	*p*
	**ELISA**											
in serum	284	16	1	0.001	275	25	10	0.009	273	27	12	0.013
in urine	256	44	29	0.201	239	61	46	0.943	243	57	42	0.722
	**Luminex**											
in serum	279	21	6	0.004	253	47	32	0.286	258	42	27	0.155
in urine	242	58	43	0.776	239	61	46	0.943	246	54	39	0.570

**Table 3:** Spearman’s rank correlation coefficients between creatinine, estimated glomerular filtration rate (eGFR), and trefoil factor 3 (TFF3) levels in patients (*n* = 19), including men (*n* = 10) and women (*n* = 9) one to two days, six months, and one year after kidney transplantation (R, rho, ρ; significance level *p* = 0.05).

TFF3 Level in	Creatinine						eGFR					
	Patients after Kidney Transplantation						Patients after Kidney Transplantation					
	Total		Men		Women		Total		Men		Women	
	R	*p*	R	*p*	R	*p*	R	*p*	R	*p*	R	*p*
	**One to two days after kidney transplantation**											
serum(ELISA)	0.22	0.367	0.44	0.200	−0.30	0.433	−0.19	0.438	−0.48	0.159	0.48	0.194
urine (ELISA)	0.52	0.022	0.61	0.060	0.78	0.013	−0.63	0.004	−0.59	0.072	−0.88	0.002
serum (Luminex)	0.61	0.006	0.59	0.074	0.30	0.433	−0.63	0.004	−0.63	0.052	−0.41	0.273
urine (Luminex)	0.34	0.156	0.42	0.229	0.72	0.030	−0.45	0.053	−0.40	0.249	−0.83	0.006
	**Six months after kidney transplantation**											
serum (ELISA)	0.27	0.268	0.14	0.700	0.47	0.205	−0.34	0.152	−0.22	0.533	−0.45	0.222
urine (ELISA)	0.23	0.340	0.10	0.789	0.45	0.224	−0.21	0.387	−0.10	0.777	−0.58	0.104
serum (Luminex)	0.21	0.385	−0.01	0.973	0.67	0.050	−0.50	0.031	−0.22	0.533	−0.71	0.032
urine (Luminex)	0.11	0.657	−0.02	0.947	0.28	0.460	−0.25	0.295	−0.02	0.960	−0.45	0.222
	**One year after kidney transplantation**											
serum (ELISA)	0.15	0.528	−0.19	0.603	0.57	0.112	−0.27	0.260	0.41	0.244	−0.80	0.010
urine (ELISA)	0.24	0.314	0.18	0.627	0.25	0.516	−0.21	0.387	−0.02	0.960	−0.25	0.516
serum (Luminex)	0.23	0.349	−0.22	0.533	0.73	0.025	−0.18	0.459	0.43	0.214	−0.85	0.004
urine (Luminex)	0.19	0.437	−0.05	0.881	0.30	0.433	−0.13	0.598	0.19	0.603	−0.28	0.460

**Table 4:** Creatinine, estimated glomerular filtration rate (eGFR), and trefoil factor 3 (TFF3) concentrations in serum of men (*n* = 10) and women (*n* = 9) one to two days, six months, and one year after kidney transplantation assessed using ELISA and Luminex ((Med., median; Q_1_, lower quartile; Q_2_, upper quartile).

Level in		TFF3 (ng/mL) Med (Q_1_–Q_2_)					
		Men			Women		
		Time after Transplantation					
		One to Two Days	Six Months	One Year	One to Two Days	Six Months	One to Two Years
ELISA	serum	69.22 (30.48–87.76)	14.80 (13.29–24.89)	12.69 (8.18–17.81)	63.65 (52.04–76.98)	18.34 (12.76–22.89)	18.72 (15.32–20.92)
	urine	137.0 (66.62–266.0)	138.5 (34.91–333.5)	89.27 (49.52–290.8)	213.8 (172.6–273.9)	53.47 (45.21–212.5)	184.4 (68.60–212.9)
Luminex	serum	14.04 (9.63–22.28)	3.24 (2.71–5.98)	3.90 (2.23–6.02)	9.56 (8.24–13.95)	4.61 (2.89–8.11)	5.54 (3.26–6.97)
	urine	28.78 (14.01–41.06)	50.69 (12.77–64.59)	36.43 (13.87–83.27)	57.25 (25.14–72.29)	22.20 (18.24–76.40)	52.67 (24.02–78.24)
**Level in**		**Creatinine (mg/dL) in serum** **Med (Q_1_–Q_2_)**					
		**Men**			**Women**		
		**Time after transplantation**					
		**One to two days**	**Six months**	**One year**	**One to two days**	**Six months**	**One year**
serum		5.36 (3.31–9.04)	1.47 (1.18–1.63)	1.40 (1.22–1.54)	3.81 (2.58–4.05)	1.35 (1.03–1.48)	1.21 (1.17–1.58)
		**GFR (in mL/min/1.73)** **Med** **(Q_1_–Q_2_)**					
		**Men**			**Women**		
		**Time after transplantation**					
		**One to two days**	**Six months**	**One year**	**One to two days**	**Six months**	**One year**
estimated		11 (7–19)	58 (47–69)	59 (52–66)	14 (12–21)	47 (41–57)	49 (39–54)

**Table 5:** Differences in values in serum and urine between two measurements (A–B, one to two days to six months; A–C, one to two days to one year; B–C, six months to one year; U, Mann-Whitney test value for small-size groups; *p*, significance level *p* = 0.05).

Measurements	Sum of Ranks (ELISA)	Sum of Ranks (Luminex)	U Mann-Whitney	
			U	*p*
A–B TFF3 in serum	495	246	56	<0.0001
A–C TFF3 in serum	477	264	74	0.002
B–C TFF3 in serum	381	360	170	0.770
A–B TFF3 in urine	397	344	154	0.448
A–C TFF3 in urine	408	333	143	0.280
B–C TFF3 in urine	386	355	165	0.661

**Table 6:** Demographic data of the study group (M, men; W, women).

Patient Number	Gender	Age at tx (in Years)	Weight (in kg)	Total Dialysis Time (in Years)	Place of Residence	Diagnosis of the Disease
1	M	49	66	16	village	Primary glomerulopathies without renal biopsy
2	W	40	61	5	village	Secondary glomerulopathies—in systemic lupus erythematosus
3	W	58	80	2	city with a population of over 100,000	Diabetic nephropathy—in type I diabetes
4	M	55	72	2	village	Primary glomerulopathies with renal biopsy
5	M	50	82	0	village	Primary glomerulopathies with renal biopsy
6	W	63	71	1	city with a population of over 100,000	Primary glomerulopathies with renal biopsy
7	M	60	80	3	town with less than 20,000 inhabitants	Hypertensive nephropathy
8	M	54	91	3	city with a population of over 100,000	Hypertensive nephropathy
9	M	36	105	1	city with a population of over 100,000	Cystic kidney disease—polycystic kidney disease
10	W	71	68	0	city of 20,000–100,000 inhabitants	Condition after right nephrectomy due to roponephrosis
11	W	57	68	3	town with less than 20,000 inhabitants	Hypertensive nephropathy
12	W	47	78	0	city of 20,000–100,000 inhabitants	Interstitial non-bacterial nephritis—other or unspecified
13	M	61	88	0	town with less than 20,000 inhabitants	Hypertensive nephropathy
14	W	49	70	2	city of 20,000–100,000 inhabitants	Hypertensive nephropathy
15	W	63	75	2	town with less than 20,000 inhabitants	Interstitial bacterial nephritis—with bladder dysfunction
16	M	49	114	2	city of 20,000–100,000 inhabitants	Cystic kidney disease—polycystic kidney disease
17	M	26	70	5	town with less than 20,000 inhabitants	Primary glomerulopathies with renal biopsy—(FSGS) focal glomerular vitrification/sclerosis
18	M	67	76	5	city with a population of over 100,000	Cystic kidney disease—polycystic kidney disease
19	W	31	73	1	village	Primary glomerulopathies with renal biopsy

**Table 7:** Demographic data of the control group (M, men; W, women).

Number of Participants	Gender	Age (in Years)	Weight (in kg)	Place of Residence
1	M	28	58	city with a population of over 100,000
2	W	28	90	
3	M	33	90	
4	W	30	65	
5	W	44	75	

**Table 8:** Characteristics of patients in the study and control groups (AM, arithmetic mean; SD, standard deviation; Med., median; Min, minimum value; Max, maximum value; Q_1_, lower quartile; Q_2_, upper quartile).

	Study Group			Control Group	
	Age at tx (in Years)	Weight (in kg)	Total Dialysis Time (in Years)	Age (in Years)	Weight (in kg)
AM ± SD	51.90 ± 12.06	78.32 ± 13.33	2.79 ± 3.60	32.60 ± 6.69	75.60 ± 14.47
Med	54.00	75.00	2.00	30.00	75.00
Min	26.00	61.00	0.00	28.00	58.00
Max	71.00	114.0	16.00	44.00	90.00
Q_1_	47.00	70.00	1.00	28.00	65.00
Q_2_	61.00	82.00	3.00	33.00	90.00

## Data Availability

The data presented in this study are available on request from the corresponding author.
